# Analysis of network expression and immune infiltration of disulfidptosis‐related genes in chronic obstructive pulmonary disease

**DOI:** 10.1002/iid3.1231

**Published:** 2024-04-05

**Authors:** Yanqun Liu, Tao Zhu, Juan Wang, Yan Cheng, Qiang Zeng, Zhangqiang You, Guangming Dai

**Affiliations:** ^1^ The No. 1 Department of Gerontology The Third Hospital of Mianyang, Sichuan Mental Health Center/The Third Hospital of Mianyang (Sichuan Mental Health Center) Mianyang China; ^2^ Respiratory Medicine and Critical Care Medicine Suining Central Hospital Suining China; ^3^ Ecological Security and Protection Key Laboratory of Sichuan Province Mianyang Normal University Mianyang China; ^4^ Department of Geriatrics First People's Hospital of Suining City Suining China

**Keywords:** chronic obstructive pulmonary disease, disulfidptosis, immune response, molecular clusters

## Abstract

**Background:**

Chronic obstructive pulmonary disease (COPD) is a globally prevalent respiratory disease, and programmed cell death plays a pivotal role in the development of COPD. Disulfidptosis is a newly discovered type of cell death that may be associated with the progression of COPD. However, the expression and role of disulfidptosis‐related genes (DRGs) in COPD remain unclear.

**Methods:**

The expression of DRGs was identified by analyzing RNA sequencing (RNA‐seq) data in COPD. Further, COPD patients were classified into two subtypes by unsupervised cluster analysis to reveal their differences in gene expression and immune infiltration. Meanwhile, hub genes associated with disulfidptosis were screened by weighted gene co‐expression network analysis. Subsequently, the hub genes were validated experimentally in cells and animals. In addition, we screened potential therapeutic drugs through the hub genes.

**Results:**

We identified two distinct molecular clusters and observed significant differences in immune cell populations between them. In addition, we screened nine hub genes, and experimental validation showed that CDC71, DOHH, PDAP1, and SLC25A39 were significantly upregulated in cigarette smoke‐induced COPD mouse lung tissues and bronchial epithelial cells (BEAS‐2B) treated with cigarette smoke extract. Finally, we predicted 10 potential small molecule drugs such as Atovaquone, Taurocholic acid, Latamoxef, and Methotrexate.

**Conclusion:**

We highlighted the strong association between COPD and disulfidptosis, with DRGs demonstrating a discriminative capacity for COPD. Additionally, the expression of certain novel genes, including CDC71, DOHH, PDAP1, and SLC25A39, is linked to COPD and may aid in the diagnosis and assessment of this condition.

## Graphical abstract

This study highlights a close association between chronic obstructive pulmonary disease (COPD) and disulfidptosis, with disulfidptosis‐related genes demonstrating a discriminative capacity for COPD. Additionally, the expression of certain novel genes, including CDC71, DOHH, PDAP1, and SLC25A39, is linked to COPD and may aid in the diagnosis and assessment of this condition.

## 1 INTRODUCTION

Chronic Obstructive Pulmonary Disease (COPD) is one of the most common respiratory diseases worldwide, often closely associated with multiple risk factors such as smoking, prolonged exposure to air pollution, and genetic factors.^1^ With the continuous increase in the elderly population, COPD is imposing an ever‐growing burden on healthcare and social systems. Currently, over 3 million people worldwide die annually due to COPD.^2^ Research reports project that by 2030, COPD will become the third leading cause of death globally.^3^ Previous research has indicated that biological processes such as airway inflammation, bronchial spasms, and airway remodeling play crucial roles in its pathogenesis.^4^ Recent studies have found that COPD is closely linked to lung cancer,^5^ with some studies suggesting that COPD even increases the incidence and mortality risk of non‐small cell lung cancer.^6^ Despite significant progress in these studies, the precise etiology and pathogenesis of COPD remain incompletely understood. Therefore, a thorough exploration of the causes and mechanisms underlying COPD is of paramount importance for the accurate diagnosis and effective treatment of this disease.

In recent years, research has revealed the relationship between various cell death mechanisms and COPD. Cell death processes such as apoptosis, necrosis, ferroptosis, and pyroptosis are believed to be associated with the progression of COPD.^7^, ^8^, ^9^, ^10^ These processes typically interact within a complex network, contributing to the sustained inflammatory state observed in COPD patients. A comprehensive understanding of the specific roles of these cell death pathways and how they interplay in the pathophysiology of COPD is crucial for comprehension of this disease. For instance, Yoshida and colleagues discovered the involvement of ferroptosis in the cell death process of lung epithelial cells induced by COPD.^11^ They found that NCOA4‐mediated selective autophagy of ferritin (ferritinophagy) plays a crucial role in the degradation of ferritin, suggesting ferroptosis as a potential therapeutic target for COPD.^11^ The abnormal activation of these forms of cell death can lead to pathological changes such as lung tissue damage and aggravated inflammation. Therefore, the regulation of cell death has become a key strategy in COPD treatment.

Recent research has uncovered a novel cell death mode known as disulfidptosis, which is associated with an excessive presence of disulfide bonds in cellular proteins.^12^ Unlike apoptosis and ferroptosis, disulfidptosis occurs in an environment of glucose deficiency, particularly in cells with high expression of solute carrier family 7 member 11 (SLC7A11). This is because these cells lack a mechanism to repair disulfides, leading to disulfide stress and triggering this unique form of cell death.^13^ Current studies suggest that disulfidptosis is closely linked to alterations in the cellular redox state and can promote the death of tumor cells by altering the conformation of cellular cytoskeletal proteins.^14^, ^15^ Since cancer cells exhibit strong resistance to oxidative stress, relying on the transport of extracellular cysteine, SLC7A11 is significantly upregulated in various cancer types, including ovarian cancer, hepatocellular carcinoma, and lung adenocarcinoma.^16^, ^17^, ^18^ While disulfidptosis plays a critical role in tumor immunity and cancer therapy,^19^, ^20^ research on inducing nontumor cell death remains limited. To provide more personalized treatment for COPD patients, it is crucial to identify more suitable molecular clusters. Therefore, we can consider utilizing the genetic characteristics of disulfidptosis to identify subtypes of COPD.

This study systematically investigated the differential expression of disulfidptosis‐related genes (DRGs) and their immune characteristics in both normal individuals and COPD patients for the first time. By analyzing the expression profiles of 15 DRGs, we successfully divided 23 COPD patients into two groups and observed significant differences in immune cell populations between them. Further analysis of these differentially expressed genes (DEGs) and the pathways they are involved in was carried out using the weighted gene co‐expression network analysis (WGCNA) algorithm. Subsequently, after screening for hub genes related to disulfidptosis, we confirmed the expression of these hub genes in cell and animal experiments. Based on this, we predicted potential drugs that could modulate disulfidptosis, providing a reference for the treatment of COPD patients.

## 2 MATERIALS AND METHODS

### 2.1 Data sources

The microarray data set GSE38974 related to COPD patients is sourced from the GEO database (www.ncbi.nlm.nih.gov/geo). This data set comprises 32 samples, including 9 controls and 23 COPD patients. Additionally, the DRGs (FLNA, TLN1, PRDX1, MYH9, FLNB, ACTB, SLC7A11, RPN1, NCKAP1, NUBPL, NDUFA11, LRPPRC, OXSM, NDUFS1, and GYS1) were obtained from previous literature.^21^, ^22^ Detailed information about these genes is provided in Supporting Information: Table S1.

### 2.2 Difference analysis

Here, we conducted differential expression analysis on the data set after log transformation and standardization using the Limma R package.^23^ Significant DEGs were defined as those with |log2 fold change|≥ 1.5 and a *p* value < .05. Additionally, we employed a “Venn diagram” to identify the overlap between core module genes and DEGs. The analysis of the GEO data set was performed using R statistical environment version 4.2.2.

### 2.3 Immune infiltration analysis

Here, we conducted an analysis on the gene expression data matrix using the CIBERSORT algorithm,^24^ calculating immune infiltration scores for 22 different types of immune cells for each sample. Finally, we visualized the results using the “ggplot2” package.

### 2.4 GENEMANIA analysis

GeneMANIA is a database used for constructing protein‐protein interaction networks (http://www.genemania.org). This database showcases functional networks between genes, aiding in a deeper understanding of their functions. It provides 33 different bioinformatics research methods, including physical interactions, gene enrichment analysis, gene co‐localization, gene co‐expression, and website prediction, among others. We used GeneMANIA to generate a gene network among the DRGs.

### 2.5 Consensus unsupervised clustering of COPD patients

Cluster analysis was performed using “ConsensusClusterPlus” as described.^25^ Agglomerative PAM clustering was applied with a one‐Pearson correlation distance metric, and 80% of the samples were resampled for 10 repetitions. The optimal number of clusters was determined using the empirical cumulative distribution function (CDF) plot.

### 2.6 Functional enrichment analysis

Functional enrichment analysis was conducted with the assistance of “clusterProfiler.” KEGG enrichment analysis identified potential biological pathways and functions related to the targets, while GO analysis focused on the biological processes, cellular components, and molecular functions of the targets. The significance level of *p* < .05 was used to determine statistical significance.

### 2.7 Weighted gene co‐expression network analysis

Using the gene expression profiles, we calculated the median absolute deviation (MAD) for each gene and excluded the bottom 50% of genes with the lowest MAD. We removed outlier genes and samples using the “goodSamplesGenes” method from the R package WGCNA.^26^ Subsequently, we used WGCNA to construct a scale‐free co‐expression network. To further analyze the modules, we computed the dissimilarity of module eigen genes, determined a cut line for the module dendrogram, and merged some modules. Additionally, we merged modules with a dissimilarity of less than 0.25 to obtain the respective expression modules.

### 2.8 Prediction of potential therapeutic agents

We imported the significant hub genes into the Enrichr database^27^ (https://maayanlab.cloud/Enrichr/) to identify potential drugs for intervention. We filtered the results based on a significance level of *p* < .05.

### 2.9 Preparation of cigarette smoke extract

As previously described,^11^ approximately 30–50 mL of cigarette smoke was aspirated using a syringe and then filled with sterile phosphate‐buffered saline (PBS) in a 15‐mL BD falcon tube. We prepared a 10‐mL solution from a single cigarette. The cigarette smoke extract (CSE) solution was filtered (0.22 μm, Sartorius, 16541) to remove insoluble particles and was designated as a 100% CSE solution.

### 2.10 Animal model

This study used 8‐week‐old SPF‐grade male C57BL/6 mice (18–22 g) as the animal model. The mice were randomly divided into two groups, with six mice in each group. The COPD model was induced using cigarette smoke, as previously described.^28^ The COPD mice were placed in a smoking chamber that was designed at 50 cm*40 cm*15 cm in size. These mice were exposed to six cigarettes (each containing 10.3 mg of nicotine and 6 mg of tar, produced in China, Shishi) three times a day, for a total of 12 weeks. The control group was exposed to well‐ventilated indoor air throughout the entire experiment. After the final exposure, the mice were euthanized, and their lung tissues were collected and stored at −80°C for further analysis. All animal experiments were approved by the Scientific Research Ethics Committee of Mianyang Normal University (the ethics approval code:2023005).

### 2.11 Cell culture and treatment

Bronchial epithelial cell line BEAS‐2B (ATCC) was cultured in RPMI‐1640 medium containing 10% fetal bovine serum (GIBCO). These cells were cultured at 37°C supplemented with a 5% CO_2_ atmosphere. As previously described,^11^ BEAS‐2B cells were treated with 5% CSE for 24 h to construct a COPD cell model.

### 2.12 Quantitative real‐time PCR

Total RNA was extracted from lung tissues and BEAS‐2B cells and reverse‐transcribed into cDNA templates. Subsequently, quantitative real‐time PCR (qRT‐PCR) was performed using the CFX96 real‐time fluorescence quantitative PCR detection system (Bio‐Rad). β‐actin was used as an internal reference, and the relative expression levels of the target genes were calculated using the $2^{‐{\mathrm{\Delta \Delta C}}_{t}}$ method. The primer sequences are listed in Supporting Information: Table S2.

### 2.13 Measurement of cell viability

BEAS‐2B cells were inoculated in 96‐well plates and cells were treated with PBS or CSE for 24 h. Cell viability was subsequently determined using an MTT kit (MA0198, Meilunbio) according to the manufacturer's instructions.

### 2.14 Hematoxylin and eosin staining

Paraffin‐embedded sections of mouse lung tissue were prepared using conventional methods. The sections were then subjected to hematoxylin and eosin (H&E) staining to assess the extent of lung tissue damage.

### 2.15 Statistical analysis

Statistical analysis of our research data was performed using Prism (GraphPad Software, version 8.0). All data are presented as mean ± SD. Statistical analysis was conducted using unpaired *t* tests, as appropriate. *p* < .05 was considered statistically significant.

## 3 RESULTS

### 3.1 Molecular characterization and assessment of immune infiltration of DRG in patients with COPD

**Figure 1:**
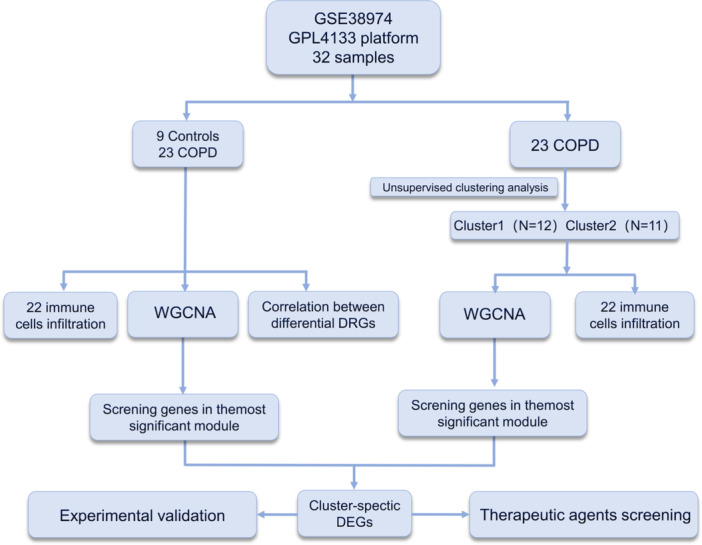
The flow‐process diagram.

To elucidate the occurrence and development mechanisms of disulfidptosis in COPD, we conducted an in‐depth analysis of GEO RNA‐seq data, including 23 samples from COPD patients and 9 samples from the normal colon control group (Supporting Information: Table S3 for details). Illustrations of the analysis workflow can be found in Figure 1.

**Figure 2:**
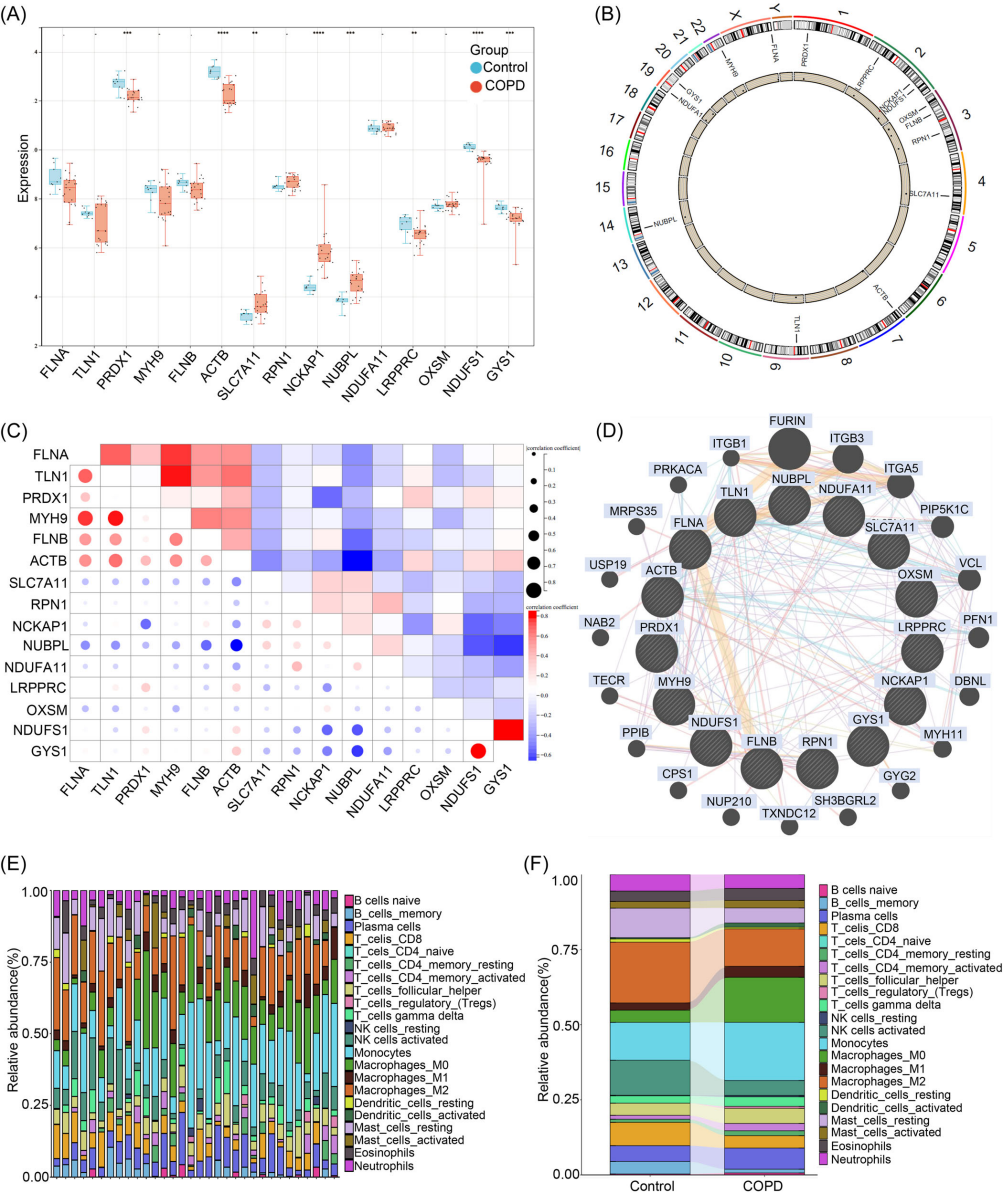
Molecular characterization and assessment of immune infiltration of DRG in patients with chronic obstructive pulmonary disease. (A) Boxplots depicting the differential expression of the 15 DRGs between the control group and COPD patients. *p < .05, **p < .01, ***p < .001, ****p < .0001. (B) The chromosomal positions of the 15 DRGs are displayed. (C) Correlation analysis of the 15 DRGs using a dot plot. (D) Network diagram illustrating the relationships among the 15 DRGs. (E and F) Show the abundance of 22 immune cell infiltrations between COPD patients and the control group. COPD, chronic obstructive pulmonary disease; DRG, disulfidptosis‐related gene.

We identified 15 genes associated with DRGs. Differential expression patterns of DRGs in the control group and COPD group were visualized through DEGs box plots, with COPD patients exhibiting high expression levels of genes such as SLC7A11, NCKAP1, and NUBPL (Figure 2A), and these results were further confirmed in a cigarette smoke‐induced COPD mouse model by qPCR (Supporting Information: Figure S1). Additionally, we displayed the chromosomal distribution of these 15 DRGs in the human genome (Figure 2B). Spearman correlation analysis revealed strong positive synergistic effects among genes encoding cytoskeletal proteins and myosin (e.g., MYH9 and TLN1), while some negative antagonistic effects were observed between SLC7A11 and RPN1 (Figure 2C). We used a circular diagram to visualize the interactions between the DRGs (Figure 2D). By employing the CIBERSORT algorithm, we successfully quantified the proportions of 22 immune cell types, revealing differences in the immune systems between COPD patients and non‐COPD individuals (Figure 2E). Interestingly, we found that in COPD patients, the levels of activated dendritic cells, M0 macrophages, resting NK cells, and activated CD4 memory T cells were relatively higher (Figure 2F), indicating a close association between the occurrence of COPD and immune system interactions. These results suggest that DRGs may play a crucial role in immune cell infiltration and molecular regulation in COPD. (Figure 2).

### 3.2 Identification of molecular clusters and comparison of immune cell infiltration characteristics in COPD

**Figure 3:**
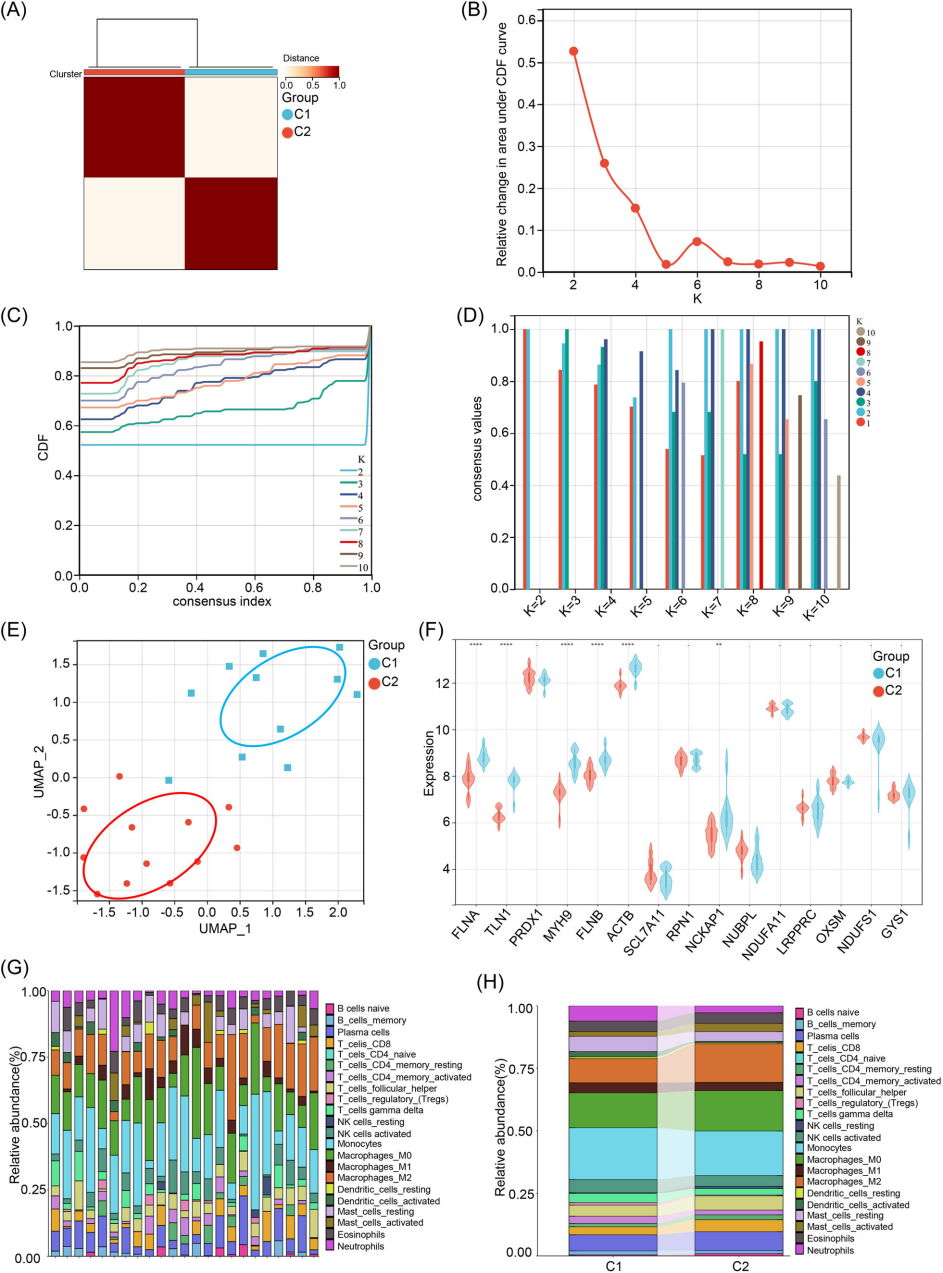
Identification of COPD clusters and comparison of immune cell infiltration characteristics. (A) Based on the expression of DRGs, COPD patient samples were divided into two clusters using the consensus clustering algorithm (*k* = 2). (B) Scores for consensus clustering. (C) Cumulative distribution function (CDF) delta area curves. (D) A heatmap of the non‐negative matrix. (E) PCA analysis illustrating the distribution of the two molecular clusters. (F) Violin plots showing the expression of 15 DRGs between cluster 1 and cluster 2. *p < .05, **p < .01, ***p < .001, ****p < .0001. (G and H) Display the abundance of 22 immune cell infiltrations between Cluster1 and Cluster2. COPD, chronic obstructive pulmonary disease; DRG, disulfidptosis‐related gene; PCA, principal component analysis.

Based on the expression data of the 15 DRGs, we employed unsupervised clustering algorithms to group the 23 COPD samples, aiming to reveal expression patterns associated with disulfidptosis. By considering various choices for the number of clusters (*k*), we determined that *k* = 2 yielded the most stable clustering results. The CDF curve was used to illustrate the degree of correlation between different *k* values, ranging from 2 to 10 (Figure 3A,B), with the change in the CDF curve ranging from 0.55 to 0.85 (Figure 3C). Further, the consistency scores for each subtype were all higher than 0.9, leading us to select *k* = 2 as the optimal molecular clustering value (Figure 3D). Additionally, Principal component analysis results also indicated significant differences between the two clusters (Figure 3E). To explore the differential expression patterns of DRGs between Cluster1 and Cluster2 and determine their molecular characteristics, we analyzed the expression patterns of these two clusters. To our surprise, the results revealed significant differences in the expression patterns of DRGs between these two clusters. Specifically, Cluster2 exhibited high expression levels of genes such as FLNA, TLN1, MYH9, FLNB, ACTB, and NCKAP1 (Figure 3F). Furthermore, we investigated the immune environment and immune cell infiltration between the two clusters. The results showed that Cluster1 had a higher proportion of Monocytes and Neutrophils, while Cluster2 exhibited a higher proportion of M2 Macrophages and activated Mast cells (Figure 3G,H). These findings contribute to a better understanding of the molecular characteristics and immune environment differences between Cluster1 and Cluster2, providing valuable clues for further research.

### 3.3 Comparison of differential gene expression signatures and pathway annotations based on clusters of disulfidptosis‐related molecules

**Figure 4:**
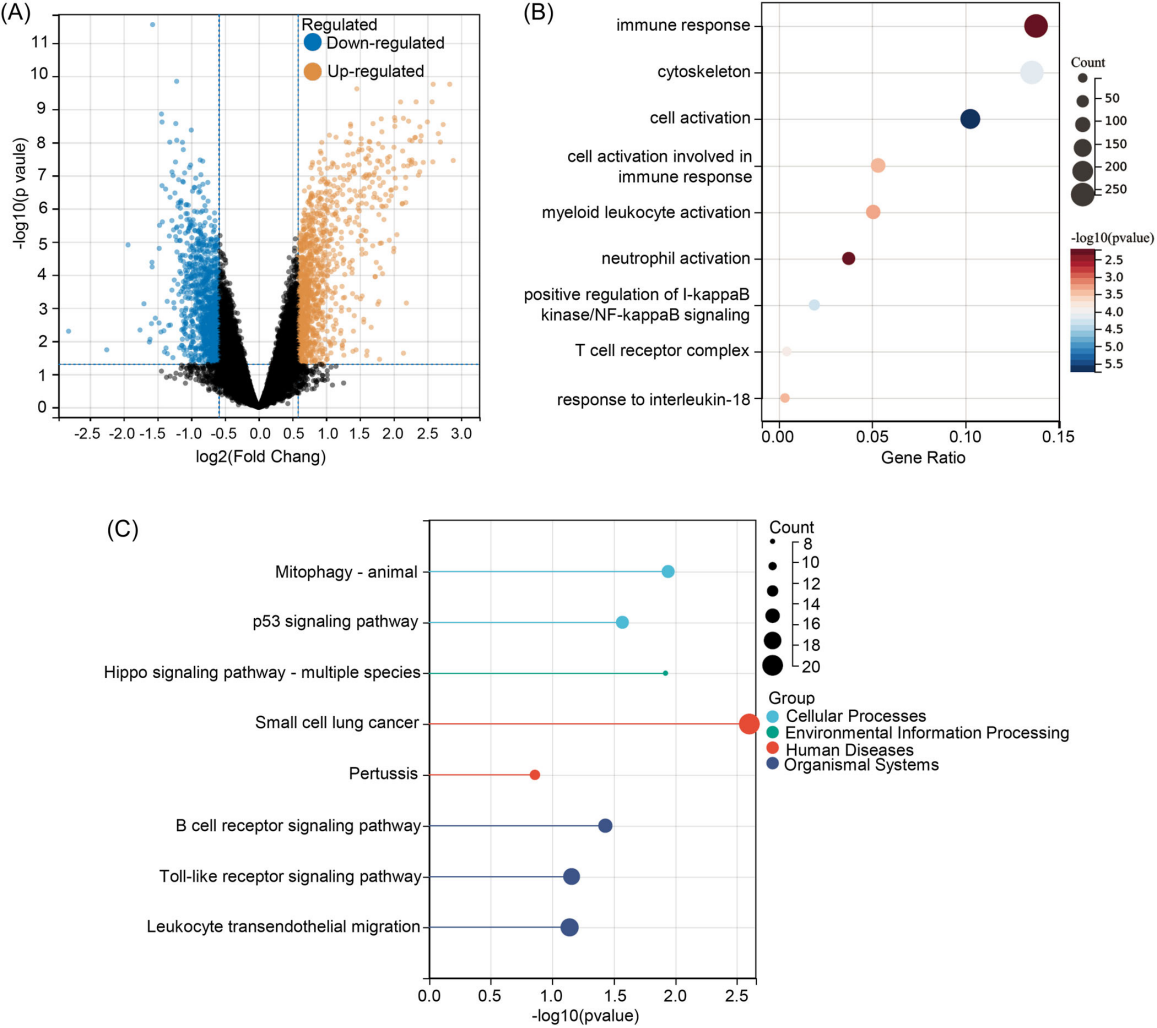
Comparison of differential gene expression patterns and pathway annotation based on the Disulfidptosis‐related molecular clusters. (A) Volcano plot of DEGs between the two DRG clusters. (B and C) GO and KEGG enrichment analysis of DEGs between the two DRG clusters. DRG, disulfidptosis‐related gene.

To uncover the distinct gene expression patterns between Cluster1 and Cluster2, we applied the following threshold filtering criteria: fold change greater than 1.5 and *p* value less than 0.05. In comparison to the Cluster1 group, a total of 146 significant DEGs were identified (Figure 4A). We visualized the expression patterns of these significant DEGs using a heatmap, providing a clear representation (Supporting Information: Figure S2). For a deeper understanding of the functions of these differential genes, we conducted GO analysis, categorizing them into biological processes, cellular components, and molecular functions. The GO analysis results indicated that these genes were primarily involved in biological processes such as immune response and myeloid leukocyte activation. In terms of cellular components, the DEGs were found to be enriched in the cytoskeleton. Moreover, in the molecular function category, there was significant enrichment in the “positive regulation of I‐kappaB kinase/NF‐kappaB signaling” (Figure 4B). Additionally, KEGG pathway enrichment analysis revealed signaling pathways associated with the disulfidptosis molecular cluster. These pathways included “Mitophagy‐animal,” “p53 signaling pathway,” “Hippo signaling pathway‐multiple species,” and “Small cell lung cancer” (Figure 4C). These results contribute to a more comprehensive understanding of the gene expression differences between Cluster1 and Cluster2.

### 3.4 Gene module screening and construction of co‐expression networks

**Figure 5:**
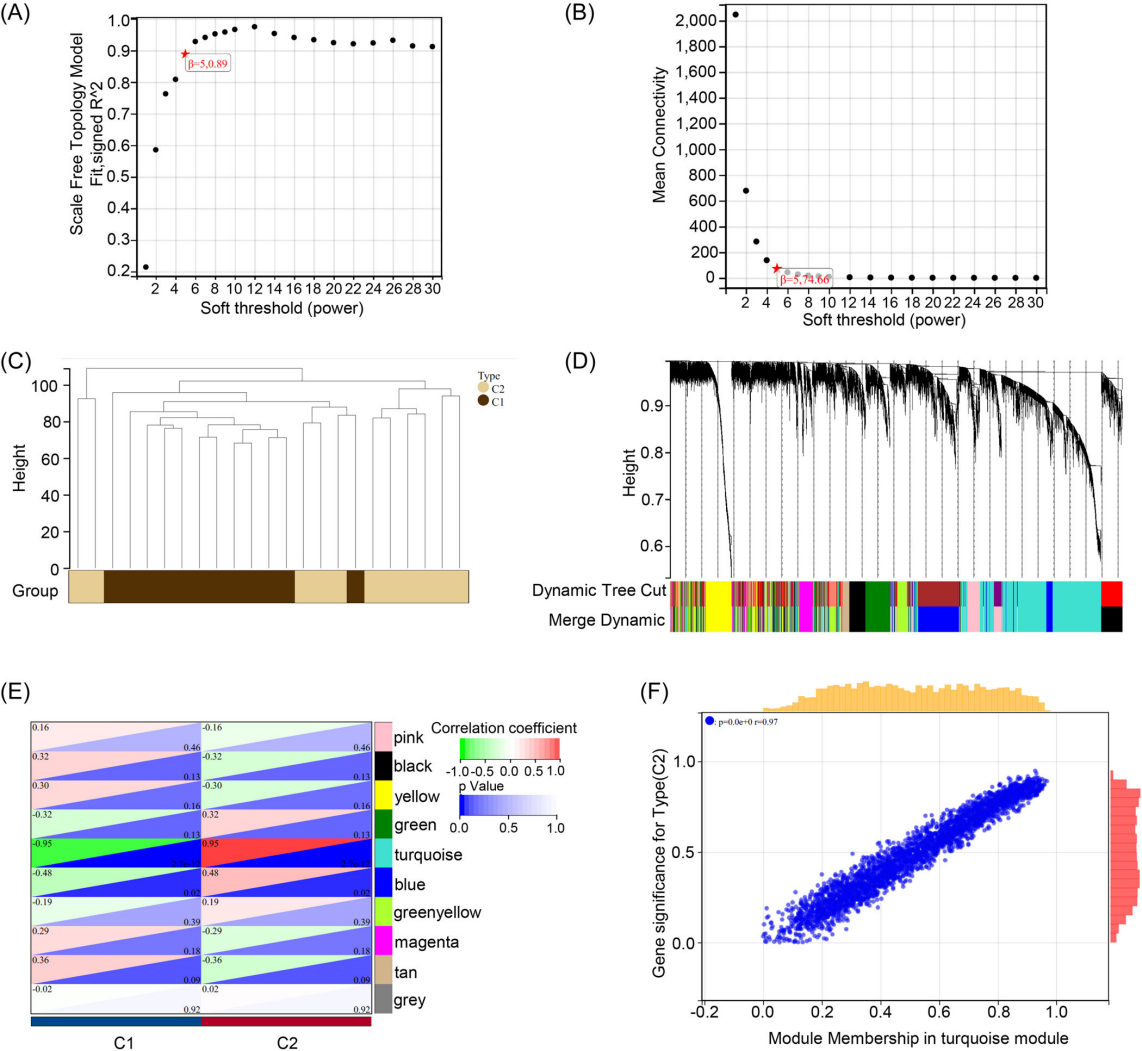
Gene module selection and co‐expression network construction based on two disulfidptosis‐related molecular clusters. (A and B) Selection of soft threshold power. (C) Classification characteristics of gene modules. (D) Dendrogram of co‐expressed genes, where different colors represent different gene co‐expression modules. (E) Module‐feature relationship heatmap, listing the associated *p* values and correlation coefficients in each cell. (F) Scatter plot showing the gene correlation of the turquoise module with the two molecular clusters.

**Figure 6:**
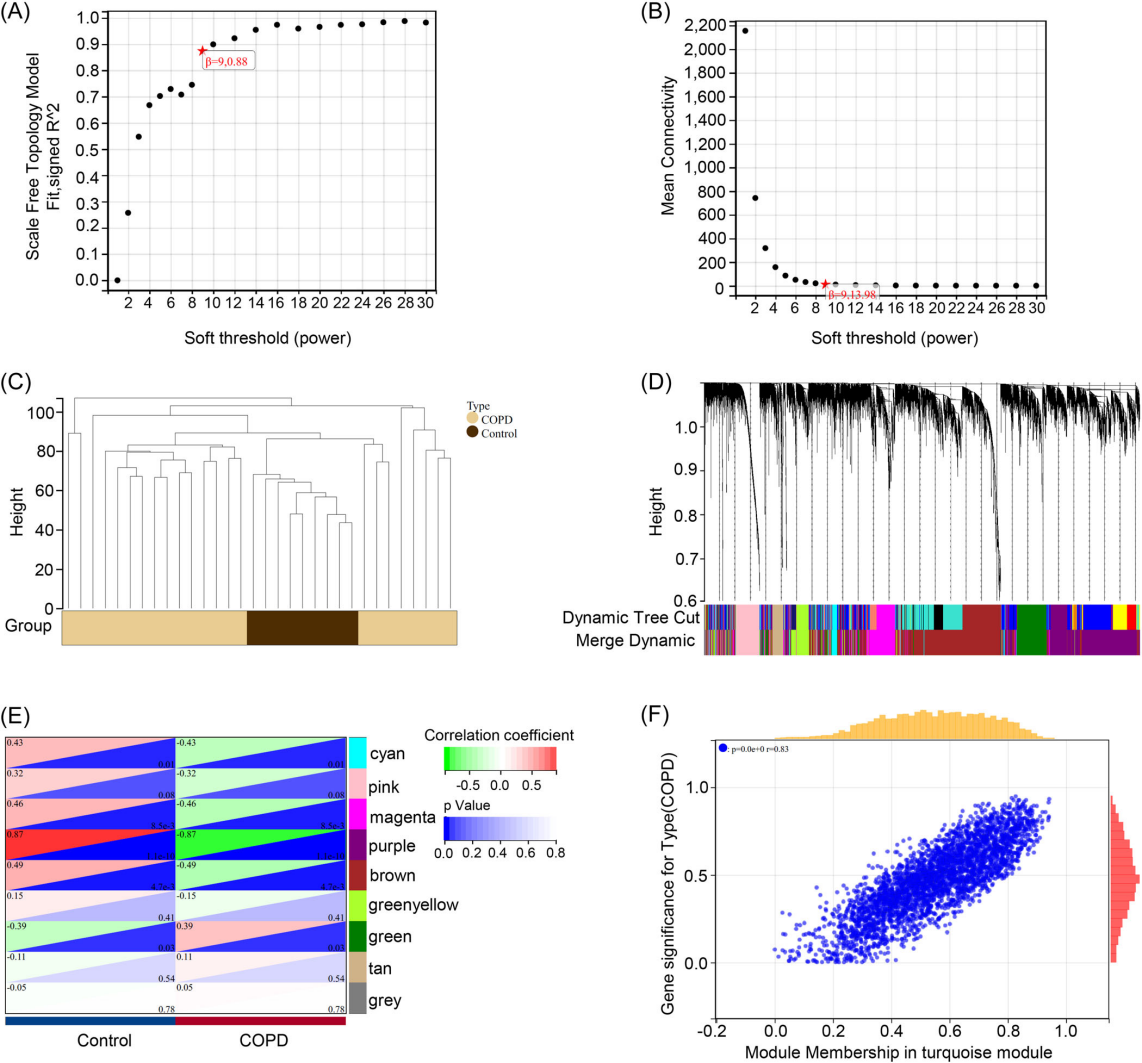
Gene module selection and co‐expression network construction based on normal and COPD patients. (A and B) Selection of soft threshold power. (C) Classification characteristics of gene modules. (D) Dendrogram of co‐expressed genes, where different colors represent different gene co‐expression modules. (E) Module‐feature relationship heatmap, listing the associated p‐values and correlation coefficients in each cell. (F) Scatter plot showing the gene correlation of the purple module with the two molecular clusters. COPD, chronic obstructive pulmonary disease.

We applied the WGCNA algorithm to analyze the genes within the disulfidptosis molecular cluster with a *β* value of 50 and an *R* ^2^ value of 0.9 (Figure 5A,B). Using optimal soft thresholding, we constructed a weighted adjacency matrix and transformed it into a TOM (Topological Overlap Matrix). Each module was randomly assigned a color, resulting in a total of 10 modules associated with the co‐expression of clinical features of the two molecular clusters (Figure 5C–E). Notably, the turquoise module exhibited significant gene‐module correlations (Figure 5F). Subsequently, we also employed WGCNA to construct a co‐expression network and modules for normal and COPD patients (Figure 6A–E). Correlation analysis indicated that the purple module genes were our prime selection, as they exhibited significant correlations with genes within the selected modules (Figure 6F).

### 3.5 Identification of hub genes

**Figure 7:**
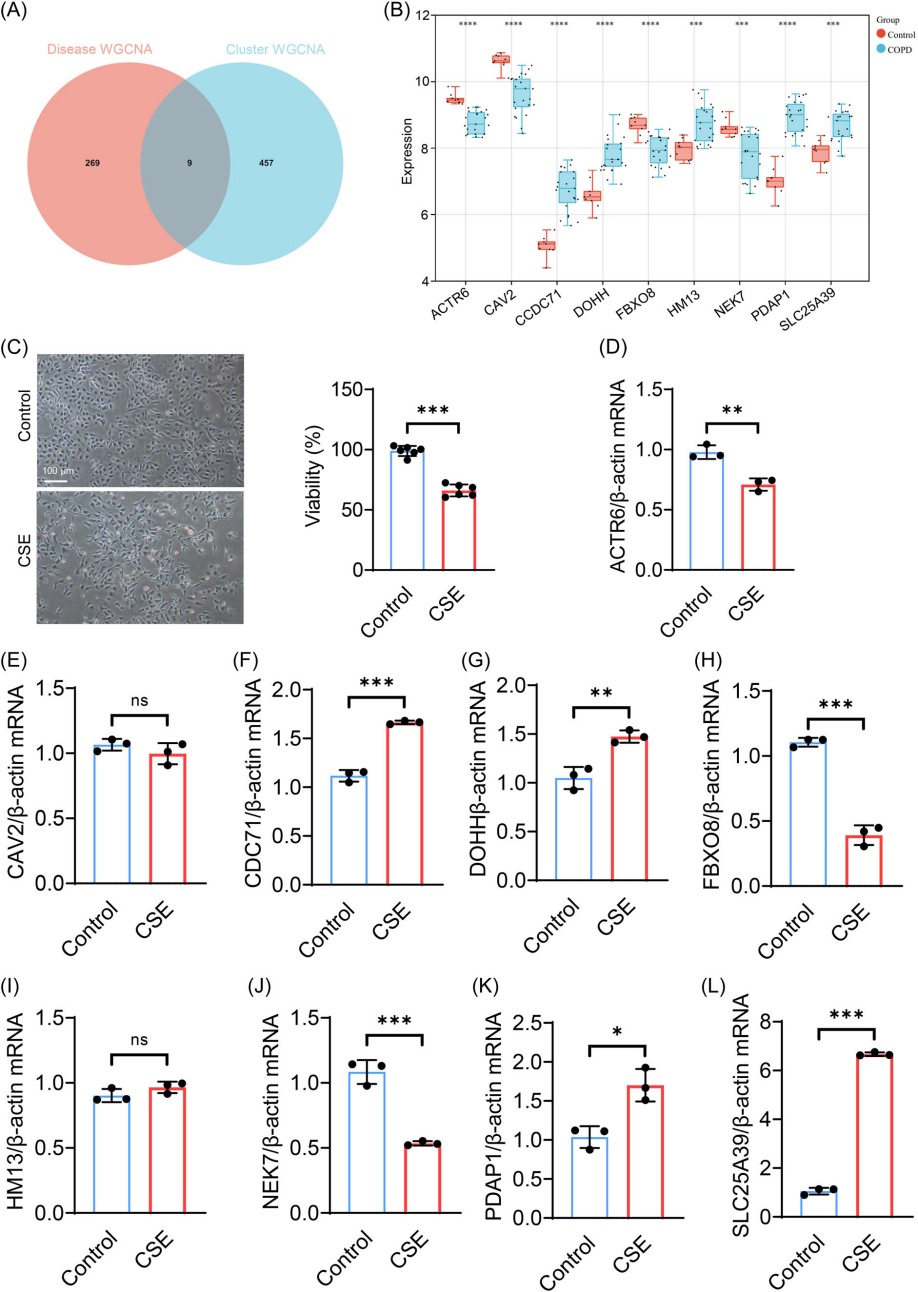
Experimental identification of hub genes in BEAS‐2B cells. (A) Venn diagram showing the intersection of hub genes between the key modules of Cluster WGCNA and Disease WGCNA. (B) Box plots displaying the expression of hub genes in COPD and control groups (GSE38974). *p < .05, **p < .01, ***p < .001, ****p < .0001. (C) Representative images of Bronchial epithelial cells (BEAS‐2B) were treated with PBS or 5% cigarette smoke extract (CSE) for 24 h, then cell viability was determined, scale bar = 100 μm (*n* = 6). (D–L) qPCR analysis of the relative expression levels of ACTR6, CAV2, CCDC71, DOHH, FBXO8, HM13, NEK7, PDAP1, and SLC25A39 (*n* = 3). COPD, chronic obstructive pulmonary disease; PBS, phosphate‐buffered saline. All data are represented as mean ± SD, *p < .05, **p < .01, ***p < .001.

**Figure 8:**
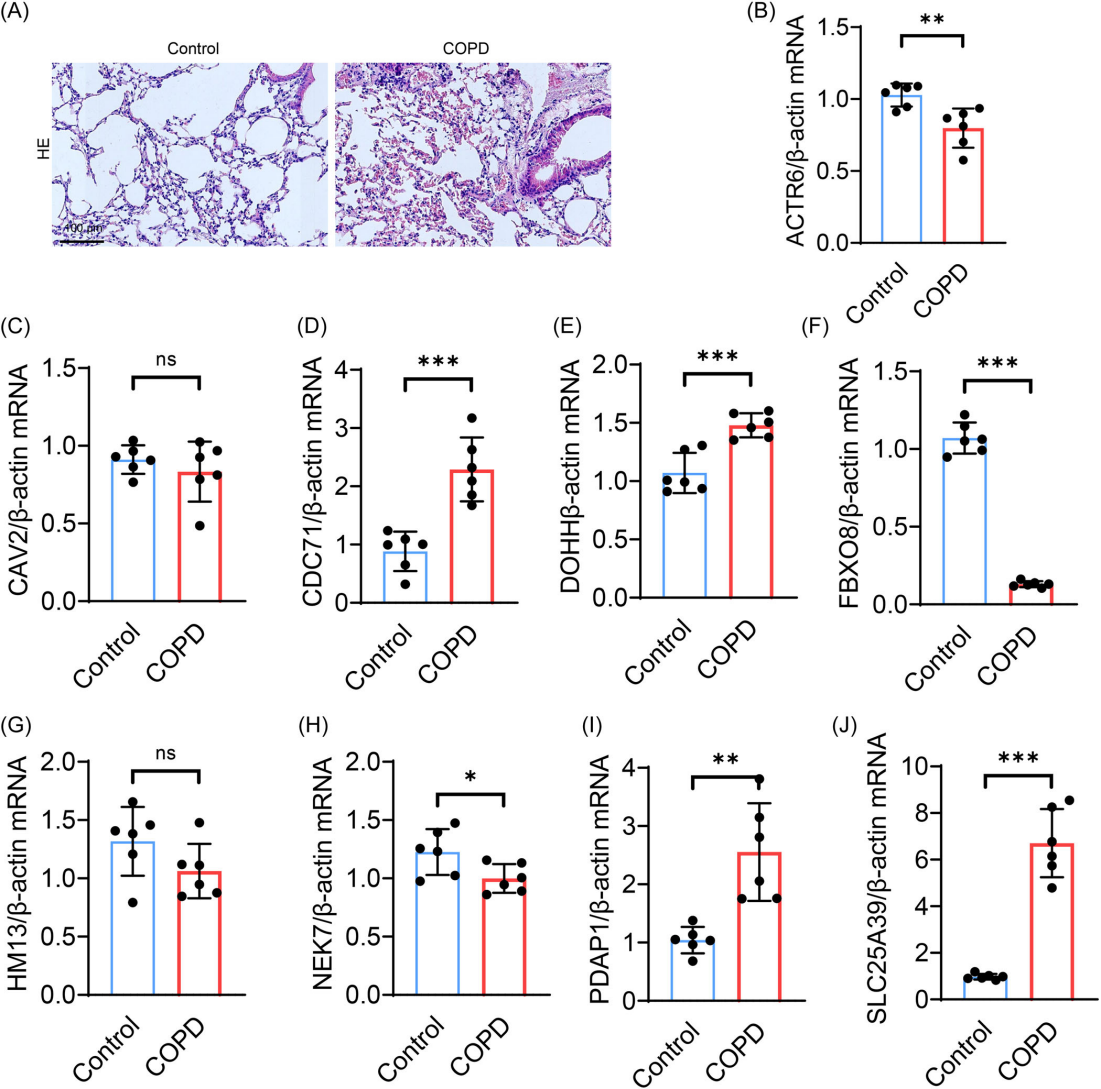
Experimental identification of hub genes in COPD mice. (A) Representative images of H&E‐stained lung tissues from control and COPD mice, scale bar = 100 μm (*n* = 6). (B–J) qPCR analysis of the relative expression levels of ACTR6, CAV2, CCDC71, DOHH, FBXO8, HM13, NEK7, PDAP1, and SLC25A39 (*n* = 6). COPD, chronic obstructive pulmonary disease; H&E, hematoxylin and eosin. All data are represented as mean ± SD, *p < .05, **p < .01, ***p < .001.

To uncover potential hub genes within the disulfidptosis molecular cluster, we employed a Venn diagram to compare the genes involved, facilitating a better examination of the overlap of target modules. We analyzed the key module genes from both Cluster WGCNA and Disease WGCNA, identifying nine specific hub genes (Figure 7A). Subsequently, we conducted an expression analysis of these nine hub genes and found that CCDC71, DOHH, HM13, PDAP1, and SLC25A39 were significantly upregulated in COPD patients, while ACTR6, CAV2, FBXO8, and NEK7 were significantly downregulated in COPD patients (Figure 7B). To further validate the expression changes of hub genes in COPD, we established an in vitro model of COPD with CSE‐induced BEAS‐2B cell injury and found that 5% CSE significantly induced cell death by MTT assay (Figure 7C). Subsequent qPCR analysis indicated that ACTR6, FBXO8, and NEK7 were significantly downregulated in the lung tissues of COPD mice, while CDC71, DOHH, PDAP1, and SLC25A39 were significantly upregulated (Figure 7D–L). In addition, we also constructed a mouse model of COPD induced by cigarette smoke. Histological observations revealed significant congestion and inflammatory cell infiltration in the lung tissues of COPD group mice (Figure 8A) and further confirmed our results by qPCR (Figure 8B–J).

### 3.6 Drugs that predict potential treatments based on hub genes

We mapped the hub genes to the Enrichr database and used a threshold of *p* < .05, we obtained potential therapeutic drugs (Table 1). Among these, compounds like Atovaquone, Taurocholic acid, and Latamoxef are potential candidates for COPD treatment.

**Table 1:** Screening of potential drugs for the treatment of COPD.

Name	*p* Value	Chemical formula	Structure
Atovaquone	.010359	C_22_H_19_ClO_3_	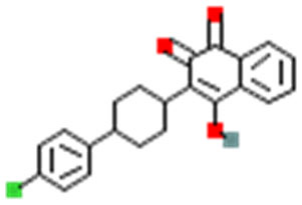
Taurocholic acid	.011196	C_26_H_45_NO_7_S	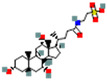
Latamoxef	.013647	C_20_H_20_N_6_O_9_S	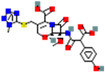
Methotrexate	.014755	C_20_H_22_N_8_O_5_	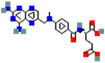
Theophylline	.017927	C_7_H_8_N_4_O_2_	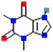
Pirinixic acid	.020963	C_14_H_14_ClN_3_O_2_S	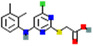
Choline	.022722	C_5_H_14_NO^+^	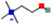
QUINOLINE	.02844	C_9_H_7_N	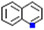
Momelotinib	.028878	C_23_H_22_N_6_O_2_	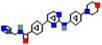
Sulpiride	.029624	C_15_H_23_N_3_O_4_S	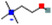

Abbreviation: COPD, chronic obstructive pulmonary disease.

## 4 DISCUSSION

In this study, we conducted the first investigation into the expression of DRGs in COPD and non‐COPD lung tissues. By analyzing the relationship between disulfidptosis and COPD, we unveiled novel and critical insights into the pathogenesis of COPD. We found that a key gene associated with disulfidptosis, SLC7A11, was significantly upregulated in COPD. SLC7A11 encodes a protein known as xCT, which is an amino acid transporter responsible for the transport of cystine and glutathione in cells.^29^ Previous studies have shown that SLC7A11 is associated with Ferroptosis in COPD.^30^, ^31^ SLC7A11 was significantly upregulated in CSE‐induced BAEC cells compared to controls, and Lin et al. demonstrated that SLC7A11 is one of the potential biomarkers for the diagnosis and treatment of cigarette smoke‐induced COPD.^30^, ^31^ Recent studies have revealed that SLC7A11 plays a crucial role in disulfidptosis, and its overexpression in cells leads to the abnormal accumulation of disulfides, resulting in protein and cytoskeletal breakdown and triggering disulfidptosis.^32^ These findings suggest that SLC7A11 is not only involved in Ferroptosis but also has a strong association with disulfidptosis in COPD.

Furthermore, the study results revealed differences in the immune cell composition of COPD patients, particularly with a significant increase in levels of infiltrating activated dendritic cells, macrophages, NK cells, and activated CD4 memory T cells. Immune cells play a crucial role in the therapeutic approaches for COPD. For instance, dendritic cell immunotherapy is an important treatment strategy that modulates and intervenes in the activity of immune cells to alleviate inflammation and control disease progression.^33^ Additionally, T cells and NK cells also play significant roles in COPD treatment, especially in combating bacterial infections and inflammation.^34^, ^35^ This suggests that the pathogenesis of COPD may be closely associated with interactions within the immune system and may involve the collaborative action of various immune cell types.

At the molecular level, this study further elucidated the expression patterns of DRGs in COPD. These genes displayed differential expression in COPD patients and exhibited distinct expression patterns in different molecular clusters. Based on the DRG cluster, we identified a total of 146 DEGs and one significant module. GO analysis revealed that these DEGs were associated with myeloid leukocyte activation and immune response. COPD is primarily an immune‐mediated common respiratory disease, where the immune system influences disease development by regulating the infiltration of immune cells in the affected areas.^36^ Furthermore, through KEGG pathway analysis, the DEGs were enriched in pathways such as Mitophagy, the p53 signaling pathway, and small cell lung cancer. Our research findings align with several other studies that have found enhancing mitochondrial autophagy through the overexpression of PRKN (parkin RBR E3 ubiquitin protein ligase) can significantly alleviate smoke‐induced COPD.^37^, ^38^ Additionally, research comparing COPD patients who smoke with those who do not smoke found significantly upregulated P53 expression in smoking COPD patients, and inhibiting the P53 signaling pathway can significantly reduce apoptosis and alleviate COPD.^39^, ^40^ Moreover, lung cancer and COPD often coexist in smokers, and the presence of COPD increases the risk of developing lung cancer.^41^

Through further experimental validation, we identified hub genes associated with Disulfidptosis, including ACTR6, FBXO8, NEK7, CDC71, DOHH, PDAP1, and SLC25A39. Among them, ACTR6 encodes a protein associated with the cell cytoskeleton, and its upregulation has been reported in non‐small cell lung cancer, potentially serving as a prognostic marker for lung cancer.^42^ In COPD, structural and functional damage to cells occurs, including destruction of alveolar walls and airway narrowing. Aberrant expression of ACTR6 may be related to these structural changes, and further research could help understand its potential role in COPD development. The FBXO8 protein is a component of the ubiquitin‐proteasome pathway used for the specific degradation of intracellular proteins.^43^ In COPD, inflammation and oxidative stress are important pathological features. This suggests that FBXO8 may be involved in COPD development by regulating the degradation of proteins associated with these processes. NEK7, CDC71, and DOHH play important roles in inflammation, cell division, and differentiation. In COPD, cell proliferation and repair abilities are impaired, and the involvement of these molecules may contribute to lung tissue repair.^44^, ^45^, ^46^ SLC25A39 protein is a transmembrane transporter protein typically involved in the transport of substances within cells.^47^ In COPD, changes in intracellular metabolism may lead to increased oxidative stress and inflammation. SLC25A39 may play a role in regulating intracellular metabolism and oxidative stress responses. These hub genes may play important roles in the development of COPD, but the specific mechanisms require further investigation.

In addition, theophylline, Latamoxef, and Methotrexate have been found to have favorable therapeutic effects in COPD.^48^, ^49^, ^50^ A study showed that the use of Methotrexate reduced the risk of COPD exacerbation.^48^ In another study, Theophylline was reported to have an inhibitory effect on airway inflammation in COPD.^51^ Furthermore, we found that especially Momelotinib its important role as a JAK1/JAK2/ACVR1 inhibitor in alleviating myelofibrosis and anti‐inflammation, which may serve as a potential drug for COPD treatment.^52^ Further research on these potential drugs is expected to reveal their exact role in the pathophysiology of COPD and provide strong support for the development of new therapeutic approaches.

In summary, this study provides new insights into the pathogenesis of COPD, particularly the role of disulfidptosis. These findings are of significant clinical importance for a deeper understanding of the pathogenesis of COPD, the identification of new therapeutic targets, and the development of personalized treatments. However, further research is needed to validate these findings and explore in greater detail the precise relationship between disulfidptosis and COPD, to better guide clinical practice.

## 5 CONCLUSION

This study highlights a close association between COPD and disulfidptosis, with DRGs demonstrating a discriminative capacity for COPD. Additionally, the expression of certain novel genes, including CDC71, DOHH, PDAP1, and SLC25A39, is linked to COPD and may aid in the diagnosis and assessment of this condition.

## AUTHOR CONTRIBUTIONS

**Yanqun Liu**: Resources; writing—original draft. **Tao Zhu**: Funding acquisition; resources; writing—original draft. **Juan Wang**: Data curation; formal analysis. **Yan Cheng**: Data curation; formal analysis. **Qiang Zeng**: Methodology. **Zhangqiang You**: Writing—review and editing. **Guangming Dai**: Conceptualization; project administration; resources.

## CONFLICT OF INTEREST STATEMENT

The authors declare no conflict of interest.

## ETHICS STATEMENT

This study obtained approval from the Scientific Research Ethics Committee of Mianyang Normal University. The current research follows the GEO data access policies and publication guidelines and strictly adheres to the principles of the “Helsinki Declaration” and the “International Ethical Guidelines for Biomedical Research Involving Human Subjects” jointly developed by the World Health Organization and the International Council for Medical Science Organizations. Conducting biomedical research involving human life sciences and medicine using human information data, ensures no harm to individuals, the exclusion of sensitive personal information, and commercial interests.

## Supporting information

Supporting information.

Supporting information.

Supporting information.

Supporting information.

Supporting information.

## Data Availability

The data that support the findings of this study are openly available in GEO at www.ncbi.nlm.nih.gov/geo/.
