# Supplementary material for: Analysis of network expression and immune infiltration of disulfidptosis‐related genes in chronic obstructive pulmonary disease
Source: Immun Inflamm Dis. 2024 Apr 5;12(4):e1231. doi: 10.1002/iid3.1231 (PMC10996381; doi:10.1002/iid3.1231)
Supplement: Supplementary file 1 — Supporting information. [file IID3-12-e1231-s005.docx]

**Supporting material**

**Analysis of Network Expression and Immune Infiltration of Disulfidptosis-Related Genes in Chronic Obstructive Pulmonary Disease**

Yanqun Liu^1#^, Tao Zhu^2#^, Juan Wang^1^, Yan Cheng^1^, Qiang Zeng^1^, Zhangqiang You^3^, Guangming Dai^4*^

**
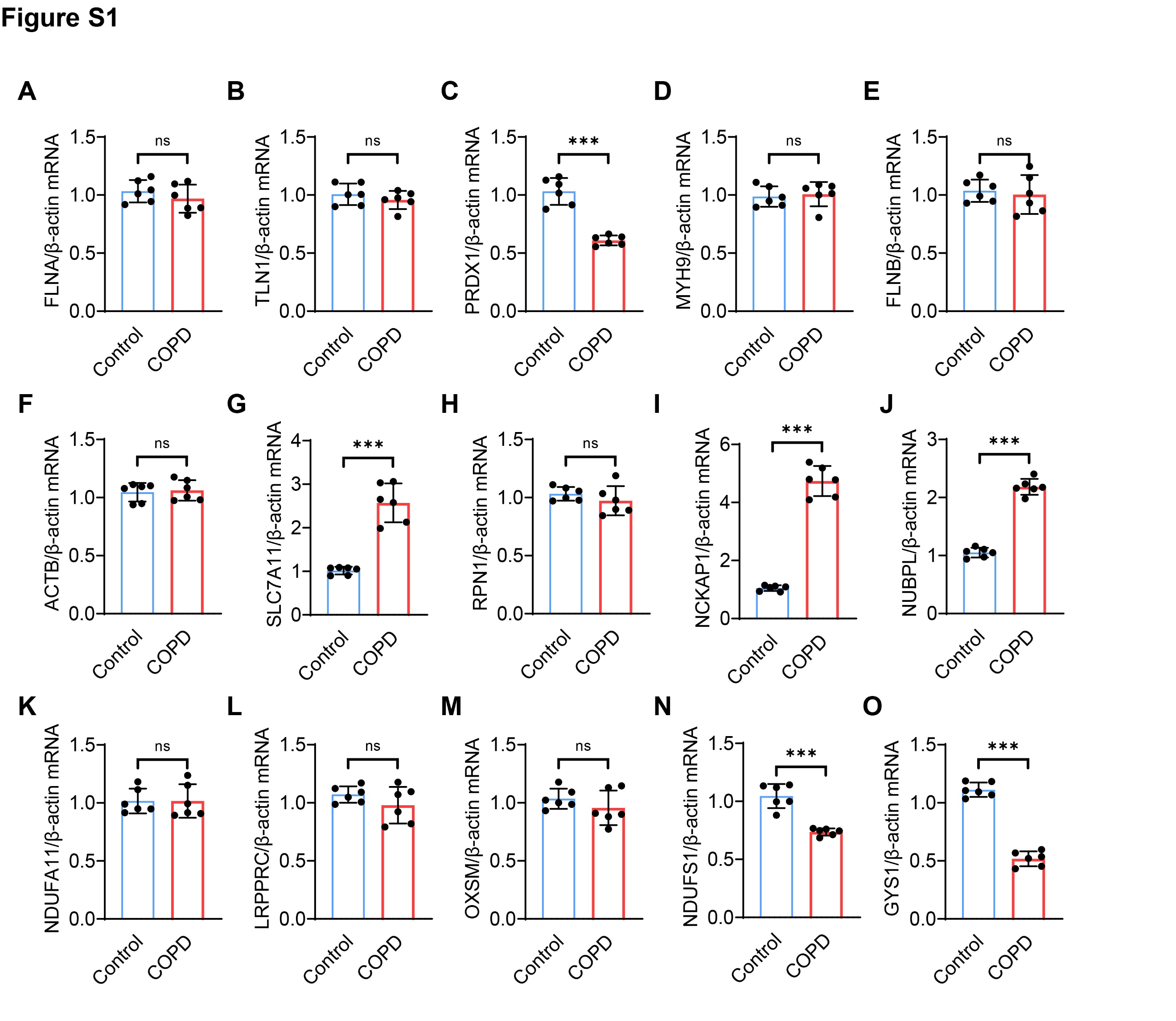
Figure S1. Expression of DRGs in COPD mice.** (A-O) qPCR analysis of the relative expression levels of FLNA, TLN1, PRDX1, MYH9, FLNB, ACTB, SLC7A11, RPN1, NCKAP1, NUBPL, NDUFA11, LRPPRC, OXSM, NDUFS1 and GYS1 (n=6). All data are represented as mean ± SD, * P < 0.05, ** P < 0.01, *** P < 0.001.

**
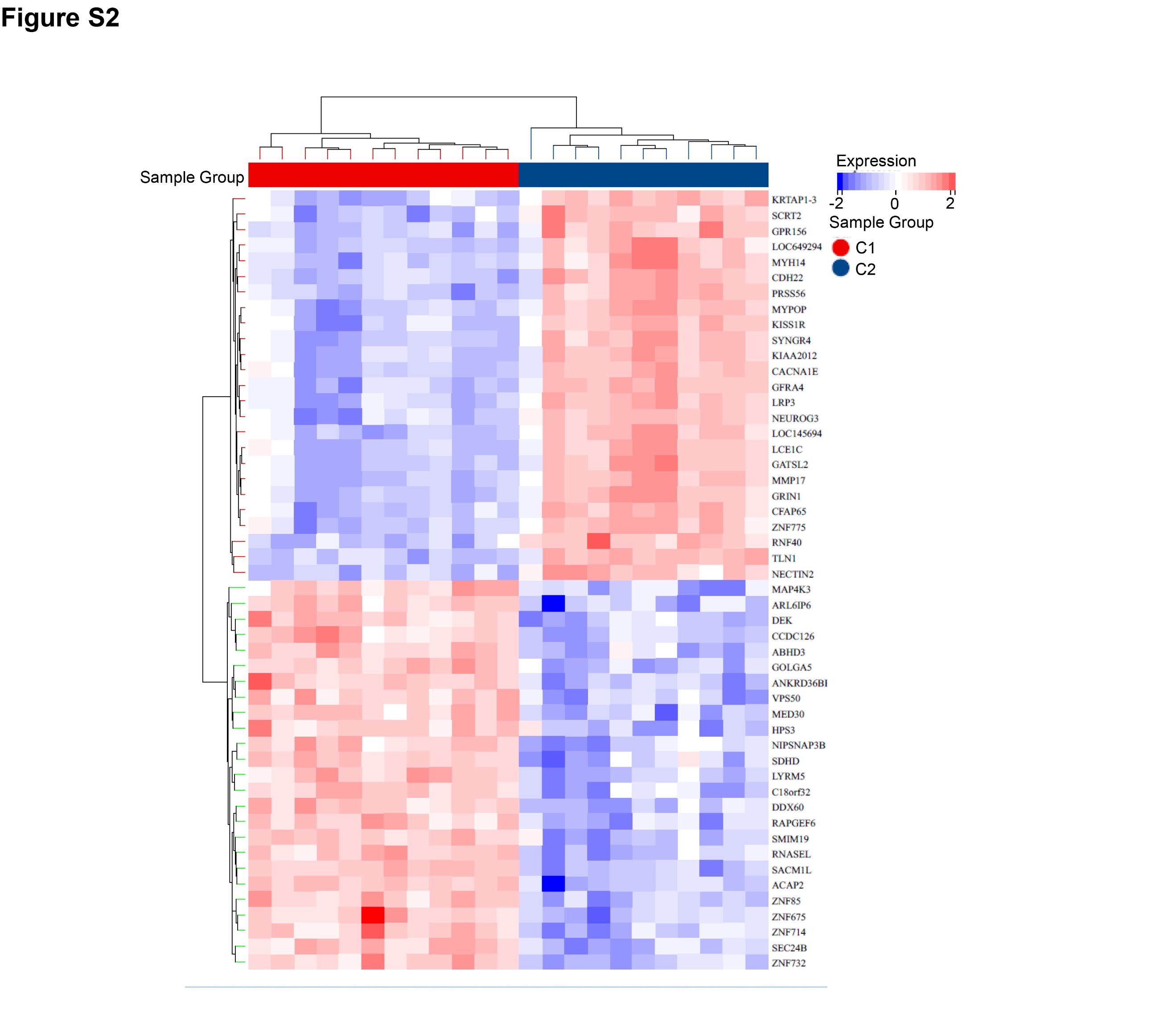
Figure S2. Comparison of differential gene expression patterns and pathway annotation based on the Disulfidptosis-related molecular clusters**. Heatmap of DEGs between the two DRG clusters.
