# Supplementary material for: Analysis of network expression and immune infiltration of disulfidptosis‐related genes in chronic obstructive pulmonary disease
Source: Immun Inflamm Dis. 2024 Apr 5;12(4):e1231. doi: 10.1002/iid3.1231 (PMC10996381; doi:10.1002/iid3.1231)
Supplement: Supplementary file 3 — Supporting information. [file IID3-12-e1231-s003.docx]

**Table S2. The primer sets for qPCR.**

| Gene | species | Forward primer (5'->3') | Reverse primer (5'->3') | Product length (bp) | |
| --- | --- | --- | --- | --- | --- |
| ACTR6 | Mouse | CAGCCATGACAGTGTCTCGT | TGTGGATCACGCAAATCGGA | | 101 |
| CAV2 | Mouse | AGAGGGGACACATGGGTTGA | AAATGGGCCCTGGGAAATAGG | | 84 |
| CCDC71 | Mouse | CCTGGGGCGATGATAGAAATGA | ACCACGAGTGCACAGCTTTC | | 148 |
| DOHH | Mouse | ATGTGCAGGACCCTACCTCT | ATGGCACGGTATCGCTCAAA | | 120 |
| FBXO8 | Mouse | TCTACATGGGGTCACTGCTC | TATCCGTGACATGACGGTGTC | | 163 |
| HM13 | Mouse | TCTCCTGTACCTGGTCCCTG | TGTGGGAAAGTGAGTGAGCC | | 139 |
| NEK7 | Mouse | GCACATTCTTTAGCTACGACAGC | ACAGTTTAGGTGCTTGCGGT | | 129 |
| PDAP1 | Mouse | CAAAAGCGAAAAGGCGTGGA | CAGATCCAGTTGCGTGACCT | | 90 |
| SLC25A39 | Mouse | AGGATCCGAGCCATGGAAATG | GGCTTTGACGTCACTACCGT | | 157 |
| FLNA | Mouse | TGTAAAGGGTCCCAGGTGAG | CCCCATGTGATGGTGACAGT | | 115 |
| TLN1 | Mouse | CCGAGCATCGAGAACTACGG | CCTCCCTTCGACACACTCTC | | 131 |
| PRDX1 | Mouse | GGGACCCATGAACATTCCCT | TAATCTCATCCACAGAGCGGC | | 179 |
| MYH9 | Mouse | ATGTGGCCTCCTCACACAAG | ATTCTTCACCGTCTTGGCGT | | 110 |
| FLNB | Mouse | CCAAGCCAGGCACCTATGTT | GACATGCATTTACCGGTGCC | | 126 |
| β-ACTIN | Mouse | ACTGTCGAGTCGCGTCCA | TCATCCATGGCGAACTGGTG | | 88 |
| SLC7A11 | Mouse | CTGCAGCTAACTGACTGCCC | TGCTATCACCGACTGGCTCT | | 146 |
| RPN1 | Mouse | GATCATCTTGCCCGAGGGAG | GCCGAACGTGTCTAGGTAGG | | 103 |
| NCKAP1 | Mouse | CAAGTTTGGAAGGCATGTGGC | TGTGCCAGCTGTTGATTGTT | | 137 |
| NUBPL | Mouse | GGTTGTGGGCGCCAGTTAT | AAGGTTCACTGCGGTGGTAG | | 171 |
| NDUFA11 | Mouse | CTCACTCAACCCCGCAGATT | ATCTGGCTTCTCTCGGACCT | | 133 |
| LRPPRC | Mouse | GACAAGGGAACAGGGACACT | AAACTAAGGACCGAGGGCGT | | 157 |
| OXSM | Mouse | TGCACAGCCTTCTTTTTACGG | GAAGTGACCAGAAGATTGCCC | | 142 |
| NDUFS1 | Mouse | GGTCCGAAGACGGGGAAAAA | TTCAACGCGTCCTCACCTTT | | 196 |
| GYS1 | Mouse | GTGTGAGGACGCAGGTAGAG | AGCTGAGGCTCCTACATCCA | | 155 |
| ACTR6 | Human | TGGTTGGTGGTGAGATGACG | CGTGCTGTTTTTGACCGGAA | | 121 |
| CAV2 | Human | ACAGCTCTTCATGGACGACG | AAAGGAGTGCGTAGTCACCG | | 169 |
| CCDC71 | Human | CTGCGCAGTGGTGATGTCTA | CTGGAGGTGATGTGGCAGTT | | 106 |
| DOHH | Human | GTCTCCAGAGCTCGGTTCTT | ATCGTGCTGTCAATGGGTCC | | 88 |
| FBXO8 | Human | TGTGCAAATCCACTTGGGGT | CCTTTGGCGAATCATCCAGG | | 174 |
| HM13 | Human | TGCATCGGTTTTCCTGTCCT | GGATTTGACTCCTCATAACTGAACA | | 77 |
| NEK7 | Human | CTCCCAACTTCCTGAGTTCTAA | AGCCCATATCCGGTCGTAAG | | 124 |
| PDAP1 | Human | AAAGAAGGTGGAGATGGGGCT | CCAGATCCAGTTGTGTGACCT | | 184 |
| SLC25A39 | Human | GCCCTTCTCAGCCCTGTACT | GACCTGGCGTTGGGTCTTTA | | 175 |
